# Enhanced Dynamic Spectrum Access in UAV Wireless Networks for Post-Disaster Area Surveillance System: A Multi-Player Multi-Armed Bandit Approach

**DOI:** 10.3390/s21237855

**Published:** 2021-11-25

**Authors:** Amr Amrallah, Ehab Mahmoud Mohamed, Gia Khanh Tran, Kei Sakaguchi

**Affiliations:** 1 Department of Electrical and Electronic Engineering, School of Engineering, Tokyo Institute of Technology, Meguro, Tokyo 152-8550, Japan; khanhtg@mobile.ee.titech.ac.jp (G.K.T.); sakaguchi@mobile.ee.titech.ac.jp (K.S.); 2 Electrical Engineering Department, College of Engineering, Prince Sattam Bin Abdulaziz University, Wadi Addwasir 11991, Saudi Arabia; ehab_mahmoud@aswu.edu.eg; 3 Electrical Engineering Department, Faculty of Engineering, Aswan University, Aswan 81542, Egypt

**Keywords:** unmanned aerial vehicles, dynamic spectrum access, quality of service, reinforcement learning, multi-armed bandit

## Abstract

Modern wireless networks are notorious for being very dense, uncoordinated, and selfish, especially with greedy user needs. This leads to a critical scarcity problem in spectrum resources. The Dynamic Spectrum Access system (DSA) is considered a promising solution for this scarcity problem. With the aid of Unmanned Aerial Vehicles (UAVs), a post-disaster surveillance system is implemented using Cognitive Radio Network (CRN). UAVs are distributed in the disaster area to capture live images of the damaged area and send them to the disaster management center. CRN enables UAVs to utilize a portion of the spectrum of the Electronic Toll Collection (ETC) gates operating in the same area. In this paper, a joint transmission power selection, data-rate maximization, and interference mitigation problem is addressed. Considering all these conflicting parameters, this problem is investigated as a budget-constrained multi-player multi-armed bandit (MAB) problem. The whole process is done in a decentralized manner, where no information is exchanged between UAVs. To achieve this, two power-budget-aware PBA-MAB) algorithms, namely upper confidence bound (PBA-UCB (MAB) algorithm and Thompson sampling (PBA-TS) algorithm, were proposed to realize the selection of the transmission power value efficiently. The proposed PBA-MAB algorithms show outstanding performance over random power value selection in terms of achievable data rate.

## 1. Introduction

The fast development of UAVs, which are commonly known as drones, has received much attention in various domains [1,2]. Recently, UAVs have been leveraged for future civil applications although their usage was restricted to military applications only during the last few years. This is considered a promising direction since UAVs have unique properties that can support this goal. UAVs are capable of various functions as they are able to fly, are maneuverable, and are easy to deploy. Hence, UAVs can handle different tasks as delivery services, traffic monitoring, aerial photography, disaster management, rescue operations, and wireless communications [1,2]. In recent years, major disasters have occurred around the world such as the great Tohoku earthquake and tsunami, which hit Japan in 2011; Hurricane Sandy on the northeastern coast of the USA in 2012; the Nepal earthquake in 2015, the massive explosion in the port of Beirut, Lebanon, in 2020; and the global wildfires in North America and Europe in 2021. All these natural disasters caused terrible damage to infrastructure and loss of human lives. The first few hours after the disaster are considered the golden relief time to provide support and emergency aid to save these precious lives. Therefore, this paper focuses on wireless communications applications for UAVs to support a post-disaster area surveillance system. Specifically, UAVs can fly over the post-disaster areas to collect live photos of the current situation and send this collected information to a disaster management center to be analyzed. This will enable rescue teams to get information promptly about the actual situation in the affected area, which will enhance their response time [3].

On the other hand, and due to the persistent increase in demand for mobile services, spectrum resources are becoming more and more scarce [4]. Therefore, it is expected that future mobile networks will host a modern communications technology that supports unsurpassed networking architecture and energy-efficient devices. To realize these novel concepts, new fundamental challenges have appeared on the surface. Unlike wired communications systems, due to the national spectrum regulations and the hardware limitation, the wireless world has limited links to distribute. Consequently, it will be mandatory for the traditional regulation of the spectrum to have a fundamental reform so that it can allow more efficient use of spectrum resources. Spectrum inefficiency has become a major concern; hence it is imperative to search for an effective solution to deal with the resource allocation problems from the spectrum and power-efficiency points of view. This solution should achieve three main goals. Firstly, it should be amenable to the distributed implementation. Secondly, it should be capable of dealing with the uncertainty caused by the lack of information. Thirdly, it should deal with users’ selfishness. One of the most promising solutions is the DSA system [5], which can be implemented as a CRN [6]. A DSA system has the ability to enhance the spectrum utilization efficiency [7]. Hence, CRN allows unlicensed Secondary Users (SUs) to coexist with the licensed Primary Users (PUs) in the licensed band without causing any harmful impact on PUs in terms of different Quality of Services (QoS) aspects. In other words, SUs can utilize a portion of the licensed PUs spectrum under certain QoS constraints [6]. Therefore, to enhance the network efficiency, SUs’ spectrum utilization should be maximized while keeping an eye on the QoS level of the high-priority traffic, i.e., the PUs traffic, to avoid any services interruption to the highly prioritized data transmission.

The concept of this resource allocation issue is considered a challenging problem for two reasons. First, the resource allocation process can be made with a large number of orthogonal communication dimensions such as time, frequency, code, space, and antenna direction [8]. Second, in order to enhance the spectrum utilization, QoS for both PUs and SUs should be maximized. To achieve this, there are different conflicting parameters that need to be jointly optimized as transmitted power, channel occupation, total throughput, and mutual interference level between simultaneous users. Therefore, for a certain number of PUs and SUs, there are indispensable targets for the optimization algorithm such as the interference threshold for each PU, the channel state information, and the geographical location for both of PUs and SUs. Moreover, this optimization scenario can be decentralized,; in other words, there is no need to deploy a fusion center to collect enough information from the environment and complete the optimization process to the end. Since energy levels are not observed in general, and both PUs and SUs form a distributed network, it can benefit from that distribution to sense the available energy at each node. From this point of view, the design of an efficient future wireless network needs to deal with the uncertainty of information besides different users’ competition and selfishness. Hence, it becomes mandatory to search for a powerful mathematical tool that can deal with such unprecedented network problems.

Machine learning (ML) algorithms, more precisely reinforcement learning (RL) algorithms, are leveraged to deal with these kinds of optimization problems [9]. The reason behind selecting RL algorithms is their capability to achieve tremendous results in generalization and efficiency, leading to their capability to tackle real-life problems, and especially in field of wireless communications [9]. Furthermore, RL algorithms are able to deal with conflicting optimization parameters of the resource allocation problem for the DSA system [10]. Without prior information about the environment, an agent can learn to enhance its future actions based on its past experience. MAB algorithms are considered one of such RL algorithms. MAB algorithms can be described as a set of actions (arms) of a bandit machine that each arm leads to a certain reward [11]. A player needs to maximize their accumulated reward over the playing epoch by choosing one arm to pull in each playing round. Moreover, this player has no idea about the reward behind each arm. So, this instantaneous reward behind each arm is revealed once the player decides to select this arm. Therefore, for this hidden setting, the player may lose some reward in each trial due to not selecting the arm that leads to the highest reward value instead of the chosen arm. This loss is denoted by regret [12]. Thus, each player should select a sequence of arms to pull to maximize their total reward over horizon, in other words, to minimize their total regret over horizon. This is a common dilemma faces MAB algorithms and it is called the exploration–exploitation trade off [13,14,15].

Over the last decade, with the rapid increase in the number of natural disasters occurring throughout the world, there has become an urgent need to develop a smart post-disaster surveillance system. This smart system should operate in a fully decentralized manner, i.e., without having a controlling center, to speed up collection and analysis of data for a post-disaster area to enhance the performance and reduce the response time of the rescue operations. DSA systems are considered a rich topic that was deeply investigated in the early 2000s for some quite old applications such as analog TV white spaces, especially in the Very High Frequency (VHF) and the Ultra High Frequency (UHF) bands [16]. Hence, we aimed to refurbish the well-known DSA system by exploiting the benefit of using ML algorithms as a modern optimization tool. Furthermore, UAVs, which are capable of flying and capturing high-resolution videos using attached cameras, were leveraged recently to support various applications in the civilian life. All these ideas motivated us to develop a smart and cheap post-disaster surveillance system by combining the advantages of DSA system, UAVs, and ML algorithms. In addition, this system is presented as unconventional method to solve the spectrum scarcity problem. In this way, DSA-system-aided ML algorithms can open the gate to unprecedented applications in the field of UAVs wireless communication networks.

In this paper, we aim to design and evaluate a spectrum allocation for a DSA system using MAB algorithms to support a post-disaster surveillance system. From a MAB perspective, UAVs, which are considered SU transmitters, will act as the player who aims to maximize their long-term reward, i.e., data rate. Furthermore, this player is constrained by a limited power budget. On the other hand, different transmitting power levels will act as arms of the bandit machine. The MAB algorithm is considered the most suitable algorithm for our optimization problem as it can deal with online optimization problems without any prior information about the environment except the player’s observations of the achieved reward while playing. Our paper adapts two different MAB algorithms, the Upper Confidence Bound (UCB) [15] and Thompson Sampling (TS) [17], to address such an optimization problem. In this paper, a modified version of MAB algorithms is proposed to treat our optimization problem. This is called the Power-Budget-Aware PBA-MAB (MAB) algorithm. The key idea behind the PBA-MAB algorithm is to include the available power budget for each UAV in the decision-making process when choosing the most appropriate transmitting power value.

From the point of view of the DSA system, the SU network, which consists of UAVs and temporary base stations, shared the spectrum resources as a CRN with the PU network, which consists of highway Electronic Toll Collection (ETC) gates and cars passing these ETC gates, under certain QoS constrains. Hence, SU transmitters are allowed to send their data without causing a harmful interference to the most precious data of the PU network. It should be mentioned that our design allows both the PU network and the SU network to coexist at the same time under a certain signal-to-interference-plus-noise ratio (SINR) threshold. Furthermore, we need to utilize the multi-objective formulation. Given the location of each PU and the power budget of each SU, we seek to design for a joint optimization problem considering different conflicting objects such as interference coordination, sum-rate maximization, and total number of active SUs in the network, subject to QoS constraints for both PUs and SUs. Despite the adversarial problem definition and the selfish behavior of each UAV toward achieving its maximum data rate, modified MAB algorithms learn how to select the most suitable action over time to enhance the overall system performance as discussed in [18,19,20] and illustrated in our paper. The main contributions of this paper can be summarized in the following points:

- The selection of the transmitted power value for UAVs aiding a post-disaster area surveillance system is formulated as an optimization problem aiming to maximize the achievable data rate while considering the limited available power budget for each UAV. This is done in a decentralized manner as there is no exchange of information among UAVs.
- Integrating the post-disaster surveillance system as a CRN is considered an unconventional solution for the spectrum scarcity problem. Furthermore, it can reduce the overhead cost of renting dedicated frequency channels for post-disaster surveillance operations, while they are rarely used just when a disaster occurs.
- Despite the nature of original MAB algorithms to maximize the long-term reward, i.e., the achieved data rate, MAB algorithms are modified to take into account the limited power budget for transmission. Therefore, the selection of the transmitted power not only aims to maximize the data rate for the current channel but also considers the remaining power budget to maximize the data rate for the next available channel.

The rest of the paper is organized as follows. Section 2 overviews the related work. Section 3 introduces the system model and the power value selection optimization problem. Section 4 introduces proposed PBA-MAB algorithms and how these algorithms can deal with this kind of optimization problem. Section 5 gives simulation and analysis of the proposed optimization scenario. Finally, we summarize the result and point out the future research in Section 6.

## 2. Related Works

Since the early 21th century, the idea of DSA gained increasing attention, especially in the US and Europe, due to the spectrum congestion [21]. An overview of the major technical and regularity issues of DSA systems was presented in [21]. The authors of [22] introduced the concept of multi-dimensional spectrum sensing and discussed the challenges associated with it. They developed prediction algorithms based on the past multi-dimensional spectrum utilization information to predict the future usage of the spectrum. With the aid of the DSA system, CRN can be established to support different applications as public safety, smart grid, broadband cellular, and medical applications. Ref. [23] discussed some challenges that faced the practical application toward this idea. An overview of CRN design layers, such as the physical layer (PHY), the medium-access control layer (MAC), and the network layer, is presented in [24]. Furthermore, the authors showed how these layers can interact with each other. The authors of [25] investigated the throughput improvements in a CRN using different channel selection techniques such as frequency hopping, frequency tracking, and frequency coding. Ref. [26] investigated the CRN formed by the incorporating radio capabilities of a Wireless Sensor Network (WSN). It addressed both advantages and limitations of CRN for WSN in conjunction with the existing applications and techniques. A continuous-time Markov chain model is implemented in [27] for a DSA system in an open spectrum wireless network. The authors of [28] examined how CRN devices can find an available spectrum channel under different system capabilities, spectrum policies, and environmental conditions. They defined this problem as a “rendezvous” problem. With the aid of RL algorithms, the authors of [29] proposed a framework for Internet of Things (IoT) devices to capture and model the traffic behavior of short-time spectrum occupancy in order to determine the existing interference in the shared bands. In [30], a novel information and energy cooperation method were introduced for cognitive Heterogeneous Networks (HetNets). This method aimed to enhance energy efficiency by solving an energy efficiency maximization problem with respect to joint time allocation and power control. The authors of [31] proposed an enhanced fusion center rule for soft decision cooperative spectrum sensing using energy detection to mitigate the noise uncertainty effect and to enhance the sensing performance of CRNs.

In recent years, there have been research efforts for using UAVs to support post-disaster area applications. In [32], the authors used UAVs with conjunction with cellular network and WSN to aid disaster management applications. A genetic algorithm was used in [33] for UAV location optimization to enhance the overall coverage and data rate of the wireless network. The authors of [34] proposed an effective method to support rescue operations in locating victims of a natural disaster. This was done with the aid of lidar and infrared depth cameras attached to UAVs to build a detecting system independent of the illumination intensity. A video recorder and a geolocation module attached to UAV were used in [35] to search for survivors in a post-disaster area. In [36], the authors examined flying communication services using Wi-Fi, video camera, and web servers attached to UAVs. They aimed to enable affected users after a disaster to use their smartphones for texting and video communication in real-time. The authors of [37] proposed a mobility model based on self-deployment of an aerial ad hoc network based on the Jaccard dissimilarity metric for a post-disaster area. The software simulation integrates the mobility of victims and generate a corresponding UAVs mobility model to trace those victims. In [38], authors proposed an energy efficient task scheduling for the collected data by UAVs from ground IoT network to support a disaster management system.

In [39], UAVs were used as on-demand airborne relays to connect remote users with a cellular BS when they were separated by vast obstacles. Furthermore, UAVs can be used in WSNs to distribute and collect information in both of Control Plane (CP) and Data Plane (DP) from wireless sensors deployed on the ground level [40,41]. UAVs are being used to assist the management and control of Vehicle Ad hoc NETworks (VANETs) and extend its coverage [42]. All the above existing research works assume a full awareness of the network parameters, which is not the case of our paper, where there is no information change among UAVs while trying to maximize the achievable data rate, as the network is fully decentralized.

On the other hand, RL algorithms have become a promising optimization technique for solving chronic UAV problems that have occurred as a result of integrating UAVs in wireless communication applications. RL algorithms are well known for their capability to achieve near optimal results in generalization and efficiency. Therefore, they are used to tackle real-time problems in the field of wireless communications. Detailed discussion about different MAB algorithms can be found in [43,44]. It has been shown in several works that MAB algorithms can be adapted to tackle such problems related to DSA systems. The authors of [45] proposed MAB learning algorithms for CRN, and particularly for spectrum sensing in a DSA system in licensed bands [7]. Different MAB algorithms, such as UCB and TS, have been used to improve the spectrum access in unlicensed Wi-Fi networks [45,46]. The authors of [47] considered a set of policies for multiple-user-independent and identical distributed (iid) and rested MAB problems with the assumption that each SU declares its action to others, e.g., the selected channel, which is considered a strong constraint. A disputed learning and spectrum access policy for iid rewards is discussed in [48], and it was proven that this policy has a logarithmic order regret. In [49], the decentralized learning for DSA system with multiple SUs spectrum access has been studied. The authors of [50] proposed a modified MAB algorithm to solve the gateway selection in UAV wireless network for post-disaster area applications. These algorithms are considering the battery life while searching for the most suitable gateway UAV to maximize the total system throughput. A dynamic wireless channel selection based on the MAB algorithm with laser chaos time sequence is proposed in [51]. The adaptive channel selection achieved a higher throughput using four channels Wireless Local Area Network (WLAN) based on IEEE802.11a system. The authors of [52] proposed a simple and powerful tug-of-war MAB algorithm. Since this algorithm is very simple, it can be applied in wireless network selection for devices with small processing capabilities as IoT devices and smartphones. Ref. [53] studied the millimeter-wave (mmWave) two-hop relaying as a single-player MAB problem in order to enable one relay probing while maximizing the achievable spectral efficiency. This was done by using modified versions of MAB algorithms. The authors of [54] studied the problem of joint neighbor discovery and selection in mmWave device to device (D2D) networks using a stochastic budget-constraint MAB algorithm.

## 3. System Model

This section discusses the network architecture of the post-disaster area surveillance system using UAVs and the used channel model for transmitting the collected data.

### 3.1. Post-Disaster Area Surveillance System Architecture

**Figure 1:**
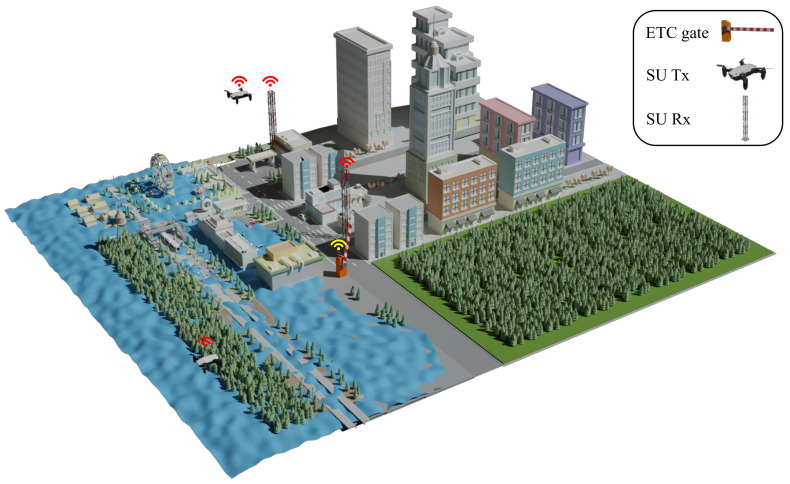
UAV surveillance-system-assisted DSA for a metropolitan post-disaster area.

Figure 1 shows a simplified version of the system architecture of the UAV wireless network in a metropolitan post-disaster area. Since the first few hours after the occurrence of the natural disaster (such as flood or earthquake) are considered the golden relief time to save human lives, as discussed in the introduction section, UAVs should collect pivotal information about victims in the damaged area using an attached high-definition camera. The collected data can be further analyzed by the disaster management center to identify victim’s exact location, number, age, gender, and injury status. On the other hand, temporary base stations are deployed in the disaster area to collect this information from surveillance UAVs and send them to the disaster management center to aid rescue teams. These temporary base stations are used as charging stations for UAVs. Furthermore, they are considered the starting flying points. UAVs fly over the disaster area to capture live photos of certain points at the damaged area. The way in which these temporary base stations transmit the collected data to the disaster management center, and the method for selecting surveillance points, are outside the scope of this paper. Moreover, we assumed in this paper that the different locations in the affected area have the same weight of importance, so these points were chosen on random bases.

On the other hand, our system aims to build this surveillance system using CRN. Therefore, the SU network, which is represented by UAVs and temporary base stations, will utilize the same frequency band of the PU network. The PU network is represented by ETC gates and bypassing vehicles in a nearby highway. In this way, we aimed to reduce the cost of reserving dedicated channels for surveillance system while it is being used during the time of natural disasters only. Each UAV collects and sends data to its corresponding temporary base station. Furthermore, each UAV should not deal harmful interference to the transmitted data between ETC gates and vehicles on the nearby highway. It should be mentioned that our optimization problem design is considered a soft-spectrum allocation. The difference between conventional spectrum allocation that have been studied in [55,56] and our optimization problem is that the conventional optimization problem treats the spectrum allocation as a hard allocation problem; i.e., no two users (PU and SU) can share same channels at the same time. However, our design introduces other orthogonal dimensions of the threshold to enable more than one user to coexist at the same frequency band if their QoS constraints are not violated. Furthermore, for the sake of generalization, we supposed that all PU channels, which connect every ETC gate and nearby vehicles which are passing this ETC gate, are always active and occupied with the PU network traffic. In this way, we considered the worst-case design scenario in which the QoS constraints should be carefully verified during the optimization process.

### 3.2. Problem Formulation

In the following, our design employs the physical model proposed in [57], which provides a path-loss model to realize the communication environment. It is assumed UAVs can communicate to temporary base station via air-to-air wireless communication link. Basically, this type of link can be called a Line of Sight (LoS) wireless communication link. Since the design is built using CRN, which shares the spectrum between PU network and SU network, this shared frequency band is split into *Q* independent sub-bands, and each sub-band has a bandwidth *W* in Hertz. Each primary and secondary transmitter receiver pair, referred to as primary and secondary users, is numbered by indices $\psi \in \Psi =\{PU_{1},\cdots ,PU_{\Psi }\}$ and $\omega \in \Omega =\{SU_{1},\cdots ,SU_{\Omega }\}$ . Hence, at any time *r*, the general path-loss formula between any transmitter α and any receiver β can be expressed by:

(1) $$L_{\alpha \beta ,q}\left(r\right)=\frac{G_{Tx,\alpha }G_{Rx,\beta }}{d_{\alpha \beta }^{\xi }}{\left(\frac{c}{4\pi f_{q}\left(r\right)}\right)}^{2}$$

where $G_{Tx,\alpha }$ and $G_{Rx,\beta }$ are the transmit and receive antenna gains, respectively, $d_{\alpha \beta }$ is the distance between α transmitter and β receiver, *c* is the speed of light, $f_{q}\left(r\right)$ is the carrier frequency of sub-band *q*, and ξ is the attenuation constant for the LoS wireless communication link. For the current design, it is assumed that the pass loss is the dominant loss factor for the received power. Hence, the effect of multi-path fading and shadowing is ignored. Furthermore, we assumed the transmitted signal is affected by an Additive White Gaussian Noise (AWGN) channel with zero mean and $N_{0}$ variance. Therefore, the SINR of SU ω in carrier *q* at time *r* can be given by:

(2) $$\gamma_{\omega ,q}\left(r\right)=\frac{p_{\omega ,q}\left(r\right)L_{\omega \omega ,q}\left(r\right)}{N_{0}+\sum_{\lambda \in \Psi \cup \Phi ,\lambda \neq \omega }p_{\lambda ,q}\left(r\right)L_{\lambda \omega ,q}\left(r\right)}$$

where $p_{\omega ,q}\left(r\right)$ and $p_{\lambda ,q}\left(r\right)$ denote the transmitted power of the ω -th SU and the λ -th PU or SU, respectively. For a successful established communication link, the SINR should satisfy a condition that the achievable SINR must be greater than the threshold SINR, which is given by $\gamma_{\omega ,q}\left(r\right)>\gamma_{\omega \mathrm{TH},q}\left(r\right)$ . Under these assumptions, the achievable data rate can be calculated by:

(3) $$R_{\omega ,q}\left(r\right)=\left\{\begin{array}{cc} W\sum_{q=1}^{Q}\log_{2}\left(1+\gamma_{\omega ,q}\left(r\right)\right),\; & \mathrm{if}\;\gamma_{\omega ,q}\left(r\right)>\gamma_{\omega \mathrm{TH},q}\left(r\right)\\ 0, & \mathrm{otherwise} \end{array}\right.$$

where *W* is the bandwidth of the communication channel.

Since the data rate is measured from the receiver side, we assumed this value is reported to the SU transmitter through a feedback channel. The concept behind this assumption comes from how modern communication systems are supposed to offer high flexibility in different ways. One of these ways is to split user and control planes to support software defined networking applications to allow flexible placement of processing function between different network nodes [58]. For PUs, it is assumed that they operate in a narrowband network, which means a pre-determined power value is assigned to each PU. This design criterion is suitable when licensed users have to operate on narrowband channels. On the other hand, for a wideband PU network, straightforward extension can be done without affecting this methodology. Since SUs need to utilize these multiband channels, where each sub-band is previously assigned to a certain PU, each SU has a power budget denoted by $P_{\max}$ . Whereas it is assumed that our PUs and SUs networks use omnidirectional antennas, the communication channel can be established according to (1) with considering antennas gain $G_{Tx,\alpha }=G_{Rx,\beta }=1,\forall \alpha ,\beta$ . Furthermore, it is assumed that each PU transmits using only single sub-band, and PUs operate in disjoint sub-bands. As a result, we have the number of PUs equal to number of channels and hence Ψ=Q . The main target of the optimization algorithm is to maximize the sum-rate, the total throughput, for the SUs network. This can be achieved by optimizing the power levels allocated for each SU within each shared traffic channel. The power allocation vector can be defined as $p_{\omega }={\left[p_{\omega ,1},\cdots ,p_{\omega ,Q}\right]}^{T}$ , where each element represents the power value for SU ω for each sub band *q*. In case that a SU has a power vector equal to zero, it means that this SU in inactive. On the other hand, for PUs, it is allowed for a single PU to transmit only on a single sub-band so that they are operating in disjoint sub-bands. Moreover, during data transmission of SUs, they should avoid causing any harmful interference to the high priority traffic that belongs to PUs network. It is mandatory for each SU to satisfy this condition and not exceed its allowed power budget during transmission as well. Considering all these power budget limitations and interference constraints, the sum-rate maximization problem can be formulated as:

(4) $$\begin{array}{c} \max\frac{1}{R}\sum_{r}\sum_{\omega }\sum_{q}R_{\omega ,q}\left(r\right)\\ s.t.\;\gamma_{\psi ,q}\left(r\right)>\gamma_{\psi \mathrm{TH},q}\left(r\right)\\ \gamma_{\omega ,q}\left(r\right)>\gamma_{\omega \mathrm{TH},q}\left(r\right) \end{array}$$

where R is the total time spent for data transmission, r=1,⋯,R , and $\gamma_{\psi ,q}\left(r\right)>\gamma_{\psi \mathrm{TH},q}\left(r\right)$ , $\gamma_{\omega ,q}\left(r\right)>\gamma_{\omega \mathrm{TH},q}\left(r\right)$ are the SINR constraint conditions for all PUs and all SUs, respectively. Thus, for SUs, it is mandatory to satisfy both SINR conditions to utilize a sub-band channel from PUs channels.

Since our network is designed in a decentralized way with no information exchange between different network elements, the only information available to UAVs are the location, the channel frequency and the transmission power of each ETC gate system. Therefore, we have developed a method to let UAVs estimate the interference caused by self-transmission and calculate the corresponding SINR value for each PU’s receiver. With the aid of Equations (1) and (2), each UAV will calculate the expected SINR value at each ETC gate under the interference effect of its own data transmission. Then, each UAV can check individually for the satisfaction of SINR conditions for both the PU network and the SU network. In such a way, there is no need to deploy a fusion center to share the SINR information between different SU network nodes, and therefore the network can be implemented in a decentralized way.

## 4. Proposed Power Budget Aware MAB Algorithm

This section discusses two proposed algorithms to tackle this rate maximization problem. These algorithms are called Power Budget Aware Upper Confidence Bound (PBA-UCB) and the Power Budget Aware Thomson Sampling (PBA-TS).

### 4.1. Proposed PBA-UCB Algorithm

UCB is considered one of the efficient MAB algorithms that can achieve balancing for the exploration-exploitation dilemma of the MAB algorithm. UCB enhances the confidence of the arm selection by decreasing the uncertainty behind the reward that will be revealed. Algorithm 1 illustrates a modified version of the UCB algorithm, which is called the PBA-UCB algorithm. This algorithm is applied to each UAV to select the most suitable transmission power in a selfish way to maximize the system rate. It is assumed that each UAV has information about the location of surrounding ETC gates operating in the surveillance area. Furthermore, they know the transmitting frequency for each ETC gate. The method of how UAVs can detect the location and the operating frequency of each ETC gate is behind the scope of this paper. Hence, each UAV tries to maximize its own data rate while competing with other UAVs to increase its transmission power while keeping an eye on the SINR threshold. At the beginning, i.e., the first N rounds, PBA-UCB algorithm, which is enabled on each UAV, tests the data rate that can be achieved by transmitting on all available channels with random transmission power and observes the achievable data rate. Afterwards, for the remaining rounds, N+1≤r≤R , the PBA-UCB algorithm picks a power value in a way that satisfies:

(5) $$p_{\omega ,q}^{*}\left(r\right)=\underset{p_{\omega ,q}\in p_{\omega }}{arg max}\left({\hat{\mu }}_{\omega ,q}\left(r-1\right)+\sqrt{\frac{\eta \ln(r)}{T_{\omega ,q}^{\left(p\right)}\left(r-1\right)}}-\frac{p_{\omega ,q}}{p_{\omega ,M}}\right)$$

where $p_{\omega ,q}\in p_{\omega }$ is the average reward obtained for transmission power value *p* in channel *q* up to the last previous round (r−1) , ${\hat{\mu }}_{\omega ,q}\left(r-1\right)$ is the average achievable data rate to the last previous round (r−1) using transmission power value *p* in channel *q*, and it can be calculated as:

(6) $${\hat{\mu }}_{\omega ,q}\left(r-1\right)=\frac{1}{T_{\omega ,q}^{\left(p\right)}\left(r-1\right)}\sum_{m=1}^{T_{\omega ,q}^{\left(p\right)}\left(r-1\right)}R_{\omega ,q}\left(m\right)$$

where $R_{\omega ,q}\left(m\right)$ is the achievable data rate, which can be obtained from Equation (3). $T_{\omega ,q}^{\left(p\right)}\left(r-1\right)$ is a count of the number of selections of this transmitting power value until the last previous round (r−1) . $p_{\omega ,q}$ is the selected power value for transmission and $p_{\omega ,M}$ is the total available power budget for UAV that can be used. This equation illustrates how PBA-UCB works. If a transmission power value is selected many times, which makes $T_{\omega ,q}^{\left(p\right)}\left(r-1\right)$ become large, the confidence bond term $\sqrt{\frac{\eta \ln(r)}{T_{\omega ,q}^{\left(p\right)}\left(r-1\right)}}$ decreases, and that causes the UAV to seek to explore other power values that are less selected in the previous rounds. On the other hand, when a transmission power value achieved a high reward, i.e., high data rate, during the past rounds, which means ${\hat{\mu }}_{\omega ,q}\left(r-1\right)$ becomes large, the UAV seeks to exploit this high-gain arm in order to achieve the maximum achievable reward during this round. Originally, the PBA-UCB algorithm sets parameter η to a positive value of 2 in most cases [13], but empirically, when it is set to η=0.5 , the performance is improved [12]. In that way, the PBA-UCB algorithm can solve the exploration–exploitation trade-off in an efficient way. Furthermore, the term $\frac{p_{\omega ,q}}{p_{\omega ,M}}$ shows how a UAV can balance between selecting a power value to achieve a high data rate and consider for the remaining power budget to be used in transmission on next available channels. It should be mentioned that this last term defines the contribution behind our proposed PBA-UCB algorithm. Since the original UCB algorithm could achieve only balancing between exploration and exploitation, our proposed PBA-UCB algorithm enables a novel way to keep an eye on the remaining power budget while balancing between exploration and exploitation. Furthermore, when selecting a transmission power, the PBA-UCB algorithm checks for the satisfaction of both PU and SU SINR conditions. Once it is satisfied, the algorithm confirms the use of this transmission power value, starts to transmit data, and calculates the corresponding rate. Otherwise, it sets the transmission power to zero and also sets the corresponding data rate to zero. In this way, the PBA-UCB algorithm can make sure there is no harmful interference that affects the PU data transmission. On the other hand, it also counts for the interference threshold on other SUs data transmission. Since the SINR condition is considered a critical design issue, this operation is done in both of the initialization phase and the rate maximization phase to ensure the feasibility of the proposed PBA-UCB algorithm. Algorithm 1 illustrates the proposed PBA-UCB algorithm.
**Algorithm 1.** PBA-UCB transmission power selection
1:**for** ω←1 to Ω **do**2:    **for** 1≤r≤N **do**                    ▹ initialization phase3:        **for** q←1 to *Q* **do**4:           Select a random value for $p_{\omega ,q}\left(r\right)$
5:           **if** $\gamma_{\psi ,q}\left(r\right)>\gamma_{\psi \mathrm{TH},q}\left(r\right)$ **then**6:               **if** $\gamma_{\omega ,q}\left(r\right)>\gamma_{\omega \mathrm{TH},q}\left(r\right)$ **then**7:                   Obtain $R_{\omega ,q}\left(r\right)$
8:                   
$T_{\omega ,q}^{\left(p\right)}\left(r\right)\leftarrow 1$
9:               **else**10:                   
$p_{\omega ,q}\left(r\right)\leftarrow 0$
11:               **end if**12:           **else**13:               
$p_{\omega ,q}\left(r\right)\leftarrow 0$
14:           **end if**15:        **end for**16:    **end for**17:    **for** r←N+1 to R **do**              ▹ rate maximization phase18:        Set $p_{\omega ,M}$ max SU Tx power19:        **for** q←1 to *Q* **do**20:           
$p_{\omega ,q}^{*}\left(r\right)=\underset{p_{\omega ,q}\in p_{\omega }}{arg max}\left({\hat{\mu }}_{\omega ,q}\left(r-1\right)+\sqrt{\frac{\eta \ln(r)}{T_{\omega ,q}^{\left(p\right)}\left(r-1\right)}}-\frac{p_{\omega ,q}}{p_{\omega ,M}}\right)$
21:           **if** $\gamma_{\psi ,q}\left(r\right)>\gamma_{\psi \mathrm{TH},q}\left(r\right)$ **then**22:               **if** $\gamma_{\omega ,q}\left(r\right)>\gamma_{\omega \mathrm{TH},q}\left(r\right)$ **then**23:                   Obtain $R_{\omega ,q}\left(r\right)$ using $p_{\omega ,q}^{*}\left(r\right)$
24:                   
$T_{\omega ,q}^{\left(p^{*}\right)}\left(r\right)\leftarrow T_{\omega ,q}^{\left(p^{*}\right)}\left(r-1\right)+1$
25:                   
${\hat{\mu }}_{\omega ,q}\left(r\right)\leftarrow \frac{1}{T_{\omega ,q}^{\left(p^{*}\right)}\left(r\right)}\sum_{m=1}^{T_{\omega ,q}^{\left(p^{*}\right)}\left(r\right)}R_{\omega ,q}\left(m\right)$
26:                   
$p_{\omega ,M}\leftarrow p_{\omega ,M}-p_{\omega ,q}^{*}$
27:               **else**28:                   
$p_{\omega ,q}^{*}\left(r\right)\leftarrow 0,R_{\omega ,q}\left(r\right)\leftarrow 0$
29:               **end if**30:           **else**31:               
$p_{\omega ,q}^{*}\left(r\right)\leftarrow 0,R_{\omega ,q}\left(r\right)\leftarrow 0$
32:           **end if**33:        **end for**34:    **end for**35:**end for**


### 4.2. Proposed PBA-TS Algorithm

TS algorithm copes with the exploration–exploitation dilemma using a different method than the previously discussed UCB algorithm. Basically, the reward gained by laying with different arms using the TS algorithm is drawn from a pure Bayesian probabilistic model [59]. In the beginning, TS uses a prior distribution for the reward based on the initialization of parameters of the probabilistic model. Afterward, it tries to keep tracking of the reward posterior distribution using the observation from the environment during the learning process. Thus, it can randomly choose a suitable arm that is matched to be optimal according to the probability model. Thus, at each round, random samples are drawn from the constructed reward’s posterior distribution. TS selects an arm to play that can maximize the selected sampled value. Then, the arm’s posterior distribution is updated by modifying its model parameters. This updated distribution will be used for the arm selection of the upcoming rounds. It is known that TS has a superb empirical performance and even better than the achieved performance of the UCB algorithm.

In our proposed PBA-TS algorithm, it is assumed that the reward, i.e., the achieved data rate, is affected by AWGN noise and mutual interference from other PUs and SUs occupying the same channel. Hence, the assumption of the Gaussian distribution is compatible with our problem formulation. The selection of the most suitable power value for transmission, which can maximize the achieved data rate, can be expressed as:

(7) $$p_{\omega ,q}^{*}\left(r\right)=\underset{p_{\omega ,q}\in p_{\omega }}{arg max}\left(\varphi_{\omega ,q}\left(r-1\right)-\frac{p_{\omega ,q}}{p_{\omega ,M}}\right)$$

where $\varphi_{\omega ,q}\left(r-1\right)$ is a sample for the previously constructed posterior distribution from the achieved data rate by a UAV ω at channel *q* with transmission power $p_{\omega ,q}$ . The posterior distribution is constructed from the Gaussian distribution $N\left({\hat{\mu }}_{\omega ,q}\left(r\right),\sigma^{2}\left(r\right)\right)$ , where ${\hat{\mu }}_{\omega ,q}\left(r\right)$ and $\sigma^{2}\left(r\right)$ are the mean and the variance of the distribution according to the model in [20], and they can be calculated as:

(8) $${\hat{\mu }}_{\omega ,q}\left(r\right)=\frac{1}{T_{\omega ,q}^{\left(p\right)}\left(r\right)}\sum_{m=1}^{T_{\omega ,q}^{\left(p\right)}\left(r\right)}R_{\omega ,q}\left(m\right)$$

(9) $$\sigma^{2}\left(r\right)=\frac{1}{T_{\omega ,q}^{\left(p\right)}\left(r\right)+1}$$

where $R_{\omega ,q}\left(m\right)$ is the achievable data rate and can be obtained from Equation (3), $T_{\omega ,q}^{\left(p\right)}\left(r\right)$ is the counted number of selections of this transmitting power value until the last previous round (r−1) , and $R_{\omega ,q}\left(m\right)$ is the achieved data rate. The term $\frac{p_{\omega ,q}}{p_{\omega ,M}}$ is deduced form the distribution to balance between the rate maximization process and the remaining power budget that should be used to transmit data over the next channels. At each round *r*, a sample $\varphi_{\omega ,q}\left(r-1\right)$ is taken from the previously constructed Gaussian distribution. Then, the optimum power value $p_{\omega ,q}^{*}$ that maximizes Equation (7) will be selected for transmission. After that, UAV ω starts to transmit over a channel *q* using $p_{\omega ,q}^{*}$ , its corresponding number of selections $T_{\omega ,q}^{\left(p^{*}\right)}\left(r\right)$ is updated, and the achievable data rate $R_{\omega ,q}\left(r\right)$ is observed to construct the Gaussian distribution for the next round r+1 . This process is conducted till the last round R . Furthermore, along with the PBA-UCB algorithm, the SINR conditions of both of PU and SU networks are examined at each time when choosing a certain power value for data transmission. If both SINR conditions are satisfied, the PBA-TS algorithm starts to use this transmission power value and counts the corresponding data rate. Otherwise, the PBA-TS algorithm sets the transmission power to zero, which leads to zero achievable data rate. The whole process of the proposed PBA-TS algorithm is summarized in Algorithm 2.

### 4.3. Complexity Analysis of the Proposed Algorithms

In this paper, we spotlight the task of UAVs to build a post-disaster surveillance system as a CRN by finding the optimal policy for each UAV. In Algorithms 1 and 2, learning processes can find the optimal transmission power value for both PBA-UCB and PBA-TS by examining various transmission power values over every channel for all UAVs using different policies. On the other hand, it tries to keep the interference level under certain thresholds. Let Ξ represent the total number of available arms, i.e., total elements of the power vector p . It is assumed that the action space is deterministic; i.e., all actions are well known to each UAV. Therefore, the number of iterations of PBA-UCB is at most of the order of O(Ω·Q·Ξ) steps. In particular, the complexity of PBA-UCB can be expressed as $O(\Omega \cdot Q^{2})$ , if the total number of the available power levels Ξ in the power vector p is equal to the total number of channels *Q*. This means the complexity of the PBA-UCB algorithm is a polynomial in Ω and *Q*. Moreover, the PBA-TS has the same computational complexity $O(\Omega \cdot Q^{2})$ as the PBA-UCB algorithm. However, the update strategy in the PBA-TS algorithm is based on sampling from the Gaussian distribution $N\left({\hat{\mu }}_{\omega ,q}\left(r\right),\sigma^{2}\left(r\right)\right)$ ; hence it may impose a slightly higher complexity depending on the sampling process.
**Algorithm 2.** PBA-TS transmission power selection
1:**for**  ω←1 to Ω **do**2:    Set ${\hat{\mu }}_{\omega ,q}\leftarrow 0,\sigma^{2}\leftarrow 1$
3:    **for** r←1 to R **do**4:        Set $p_{\omega ,M}$ = max SU Tx power5:        **for** q←1 to *Q* **do**6:           Draw a sample $\varphi_{\omega ,q}\left(r-1\right)$ from the distribution     
$N\left({\hat{\mu }}_{\omega ,q}\left(r\right),\sigma^{2}\left(r\right)\right)$
7:           
$p_{\omega ,q}^{*}\left(r\right)=\underset{p_{\omega ,q}\in p_{\omega }}{arg max}\left(\varphi_{\omega ,q}\left(r-1\right)-\frac{p_{\omega ,q}}{p_{\omega ,M}}\right)$
8:           **if** $\gamma_{\psi ,q}\left(r\right)>\gamma_{\psi \mathrm{TH},q}\left(r\right)$ **then**9:               **if** $\gamma_{\omega ,q}\left(r\right)>\gamma_{\omega \mathrm{TH},q}\left(r\right)$ **then**10:                   Obtain $R_{\omega ,q}\left(r\right)$ using $p_{\omega ,q}^{*}\left(r\right)$
11:                   
$T_{\omega ,q}^{\left(p^{*}\right)}\left(r\right)\leftarrow T_{\omega ,q}^{\left(p^{*}\right)}\left(r-1\right)+1$
12:                   
${\hat{\mu }}_{\omega ,q}\left(r\right)\leftarrow \frac{1}{T_{\omega ,q}^{\left(p^{*}\right)}\left(r\right)}\sum_{m=1}^{T_{\omega ,q}^{\left(p^{*}\right)}\left(r\right)}R_{\omega ,q}\left(m\right)$
13:                   
$\sigma^{2}\left(r\right)\leftarrow \frac{1}{T_{\omega ,q}^{\left(p^{*}\right)}\left(r\right)+1}$
14:                   
$p_{\omega ,M}\leftarrow p_{\omega ,M}-p_{\omega ,q}^{*}$
15:               **else**16:                   
$p_{\omega ,q}^{*}\left(r\right)\leftarrow 0,R_{\omega ,q}\left(r\right)\leftarrow 0$
17:               **end if**18:           **else**19:               
$p_{\omega ,q}^{*}\left(r\right)\leftarrow 0,R_{\omega ,q}\left(r\right)\leftarrow 0$
20:           **end if**21:        **end for**22:    **end for**23:**end for**


## 5. Simulation Results

In this section, the simulation results of our proposed algorithms are evaluated in terms of solution performance. We distributed each PU and SU transmitter randomly in a 5 km × 5 km area, while PUs and SUs receivers are deployed in a certain area from PUs and SUs transmitters to comply with the SINR constraint. The SINR threshold is chosen to be 30 dB for the PUs network, which is relatively high to ensure that the accumulated data transmission from SUs will not cause any harmful interference to the most valuable traffic. On the other hand, the SINR value for SUs network is set to 5 dB to ensure a successful data transmission. The transmission powers for PUs and SUs networks are set to 24 dBm and 30 dBm, respectively. We deployed 10 armed bandits to represent 10 different levels of UAVs’ transmission power. These power levels are uniformly distributed with separation equal to the maximum transmission power divided by number or armed bandits. Both PU and SU networks operate at 5.8 GHz band with a bandwidth equal to 10 MHz. Since both PUs and SUs networks operate in an open area, the attenuation constant parameter is set to 3 for a free-space communication in a metropolitan area. Table 1 summarizes the system’s parameters which are used for simulation.

**Figure 2:**
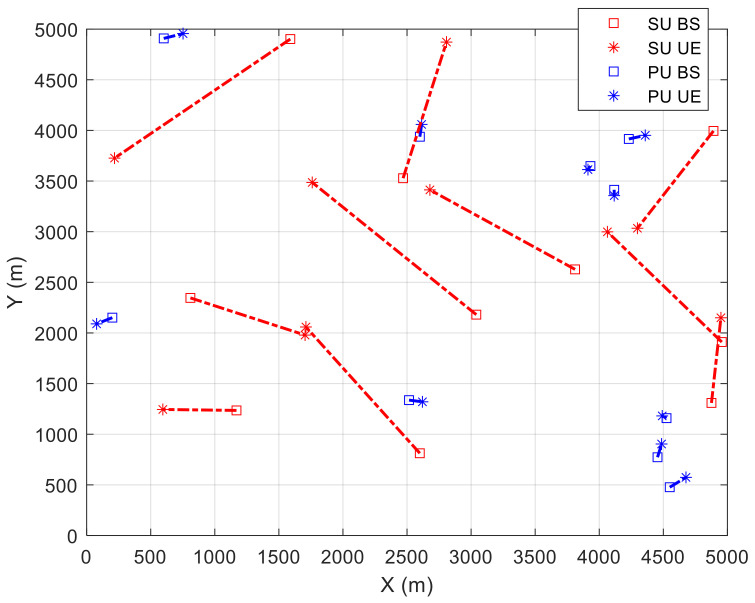
Distribution of PUs and SUs Tx/Rx pairs.

Figure 2 shows an example of PUs and SUs transmitter/receiver pairs deployment. The deployment of PU receivers, i.e., cars, in the simulation area was done in a random way within δ distance from their corresponding transmitters, while δ is chosen to achieve 30 dB at the boundary of their deployment region. The number of sub-bands is set to be equal to the number of PUs, and hence Ψ=Q , as described previously in Section 3.

### 5.1. Average Total System Rate

This section shows the performance of the total average system rate in bps/Hz against different values of UAVs and ETC gates.

**Figure 3:**
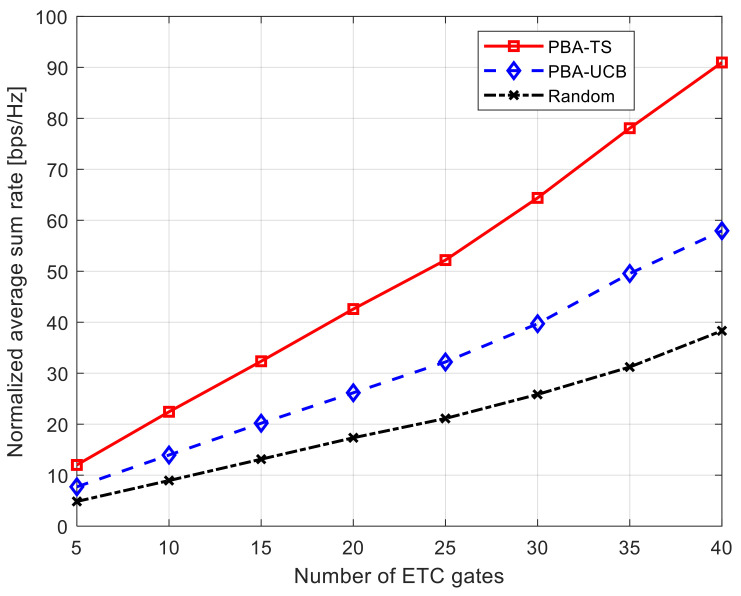
Normalized average sum rate against number of ETC gates using 10 UAVs.

Figure 3 shows the total average system rate using 10 UAVs while increasing the number of ETC gates. It is shown in this figure that the PBA-TS algorithm achieved the highest data rate performance compared to both the PBA-UCB algorithm and transmission using a random power value. The reason behind this is that PBA-TS algorithm is constructed using posterior distributions for the obtained data rates through the integrated Bayesian strategy. On the other hand, transmission using a random power value has the worst performance due to the randomness in the selection of this power value for transmission in each round. Thus, each UAV experiences random interference from not only ETC gates but also other UAVs that share these channels. Furthermore, when the number of ETC gates increases and each ETC gate has its own separate channel, the number of available spectrum resources increases as well. This leads to each UAV becoming able to transmit data over a wider band of channels and causes the total achievable average system rate to increase for both the PBA-TS algorithm and the PBA-UCB algorithm. On the other hand, and due to the randomness illustrated in this section, the increase in the achievable total average system rate using a random power value data transmission is not as high as the achievable data rate using either the PBA-TS algorithm or the PBA-UCB algorithm.

**Figure 4:**
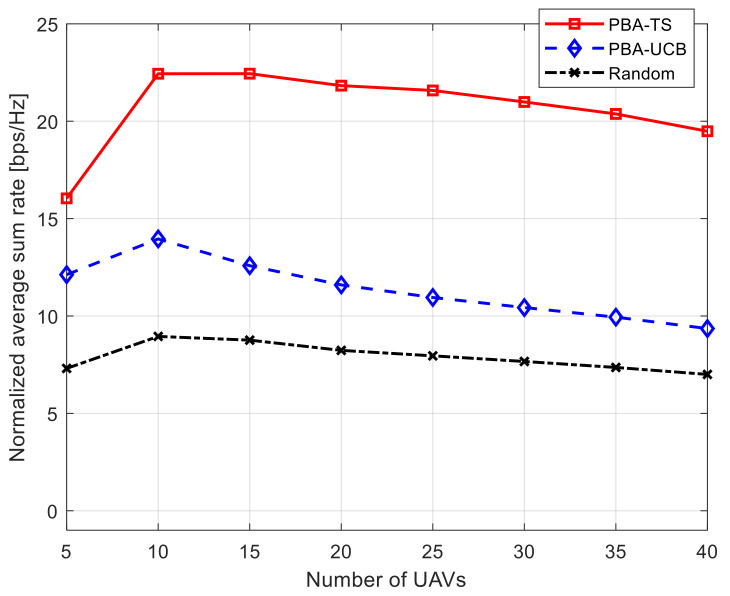
Normalized average sum rate against number of UAVs using 10 ETC gates.

Figure 4 shows the performance of the achievable total average system rate against an increasing number of UAVs while keeping the number of ETC gates equal to 10. It is interesting that at the beginning with a few increments of the number of UAVs, the achievable data rate, using our proposed PBA-MAB algorithms, is increased till a certain point. Then, the achievable data rate begins to decrease with any increment in the number of deployed UAVs. The reason behind that is that while increasing the number of UAVs, the mutual interference between UAVs increases as well. Our proposed PBA-MAB algorithms succeeded in mitigating the interference effect, which is reflected in the achievable data rate reduction. Furthermore, the proposed PBA-TS algorithm can still achieve the highest data rate performance compared to the proposed PBA-UCB algorithm and the transmission using a random power value.

### 5.2. Convergence Rate

**Figure 5:**
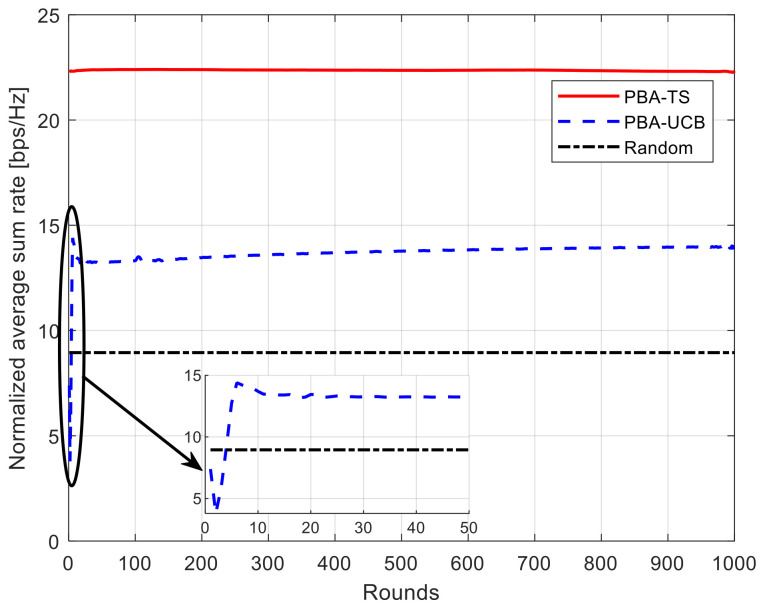
Convergence of normalized average sum rate using 10 ETC gates and 10 UAVs.

**Figure 6:**
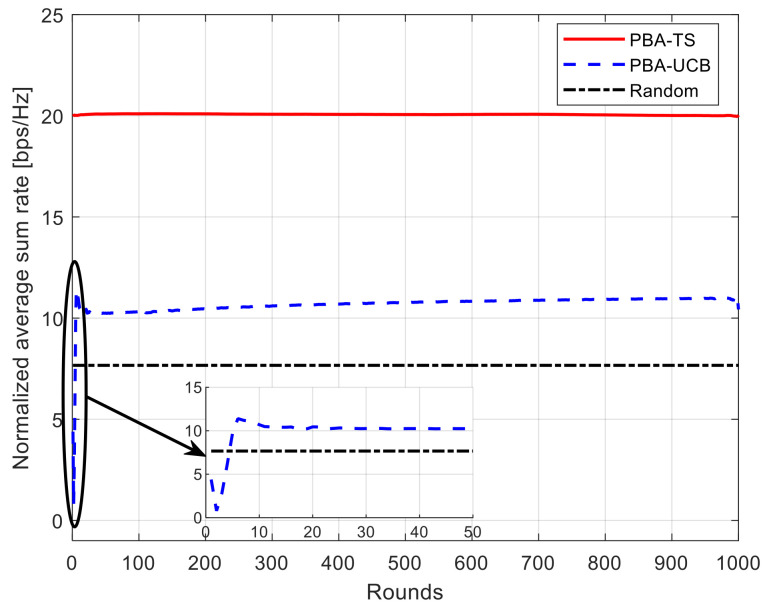
Convergence of normalized average sum rate using 10 ETC gates and 30 UAVs.

The convergence rate is considered one of the most important parameters to judge the efficiency of online learning algorithms such as MAB algorithms; the faster the algorithm can converge, the better the reward that can be gained in just a few attempts. Hence, this section studies the convergence rate of the achievable total average system rate for our proposed PBA-MAB algorithms with different settings. Figure 5 and Figure 6 show the convergence rate of the achievable total average system rate using 10 ETC gates while changing the number of UAVs to be 10 and 30. This can show the convergence rate for each algorithm under different network setup and different interference values. As shown in these figures, the horizontal axis indicates the count for rounds. Each algorithm runs its iterative process over counts till the algorithm converges toward a higher data rate. The proposed PBA-TS algorithm can converge faster than the PBA-UCB algorithm due to the fact that it uses Bayesian strategy over the posterior distributions of the reward. On the other hand, the PBA-UCB fluctuates during the few beginning rounds, and it takes more time to converge than the PBA-TS algorithm. Furthermore, it has a less convergence rate that the PBA-TS algorithm when both of the algorithms saturate by the end of the simulation rounds. These results can be concluded that both proposed PBA-MAB algorithms can deal with the adversarial network setup and selfish behavior of the UAVs. Hence, it means that every UAV learns how to select the most suitable transmission power value to enhance the overall system performance at every round. Furthermore, without loss of generality, it keeps an eye on the interference level while choosing this most suitable action.

## 6. Conclusions

In this paper, we have investigated the radio resource allocation for a CRN through DSA system to support a disaster surveillance system using UAVs wireless networks. To tackle this problem, we proposed two MAB algorithms, i.e., the PBA-UCB algorithm and the PBA-TS algorithm. The idea behind deploying MAB algorithms, as a class of RL algorithms, is the ability of MAB algorithms to solve online optimization problems with conflicting parameters that need to be jointly optimized. Since there is no information exchange between all UAVs, multi-player PBA-MAB algorithms were introduced to deal with this selfish configuration. Proposed PBA-MAB algorithms show outstanding performance over transmission using a random power value selection. Furthermore, the proposed algorithms showed a moderate convergence rate. The obtained results showed the capability of different MAB algorithms to deal with such problems with a high degree of randomness. Therefore, it can open the way for applying ML algorithms and more precise MAB algorithms to handle various wireless communication problems.

## Figures and Tables

**Table 1:** Simulation parameters.

Notation	Value
No. of armed bandits	10
Simulation area	5 km × 5 km
PU Tx power	24 dBm
$P_{\max}$	30 dBm
*W*	10 MHz
$f_{q}$	5.8 GHz
*c*	$3\times 10^{8}$ m/s
ξ	3
$\gamma_{\psi \mathrm{TH},q}$	30 dB
$\gamma_{\omega \mathrm{TH},q}$	5 dB
$N_{0}$	−100 dBm
η	0.5

## Data Availability

Not applicable.

## Abbreviations

The following abbreviations are used in this manuscript:

DSA	Dynamic Spectrum Access
UAV	Unmanned Aerial Vehicle
CRN	Cognitive Radio Network
ETC	Electronic Toll Gate
MAB	Multi-armed Bandit
PBA-MAB	Power-Budget-Aware Multi-armed Bandit
UCB	Upper Confidence Bound
TS	Thompson Sampling
PBA-UCB	Power-Budget-Aware Upper Confidence Bound
PBA-TS	Power-Budget-Aware Thompson Sampling
PU	Primary User
SU	Secondary User
QoS	Quality of Service
ML	Machine Learning
RL	Reinforcement Learning
SINR	Signal-to-Interference-Plus-Noise Ratio
WSN	Wireless Sensor Network
CP	Control Plane
DP	Data Plane
VANET	Vehicle Ad hoc NETwork
iid	independent and identical distribution
WLAN	Wireless Local Area Network
LoS	Line of Sight
AWGN	Additive White Gaussian Noise
HetNets	Heterogeneous Networks
PHY	Physical layer
MAC	Medium Access Control layer
IoT	Internet of Things
VHF	Very High Frequency
UHF	Ultra High Frequency
mmWave	millimeter-wave
D2D	Device to Device
